# Using polypropylene mesh graft for soft-tissue reconstruction in internal hemipelvectomy: a case report

**DOI:** 10.1186/1477-7819-10-124

**Published:** 2012-06-28

**Authors:** Apichat Asavamongkolkul, Saranatra Waikakul

**Affiliations:** 1 Department of Orthopaedic Surgery, Faculty of Medicine, Siriraj Hospital, Mahidol University, 2 Prannok Road, Bangkok, 10700, Thailand

**Keywords:** Internal hemipelvectomy, Chondrosarcoma, Malignant tumor, Pelvis, Polypropylene mesh graft

## Abstract

We report the case of a patient with chondrosarcoma involving the right pelvis and contralateral pubic area in a 45-year-old male who underwent an extensive internal hemipelvectomy without bony reconstruction. We demonstrate the technique of using polypropylene mesh graft for soft-tissue reconstruction. Follow-up at 7.5 years showed a good oncological and functional outcome.

## Background

Pelvic tumors have previously been treated with standard hemipelvectomy, that is, hindquarter amputation [1]. Nowadays, advances in adjuvant chemotherapy, radiation therapy, imaging and surgical techniques have allowed more patients to be treated with limb salvage surgery and have better survival rate and quality of life. However, limb salvage around the periacetabular area following malignant tumor removal is one of the most challenging procedures in musculoskeletal oncology. Because of large tumor size and the complexity of anatomy in this location, resections of tumor are mostly difficult and reconstructions are demanding procedures in this area. Recent reconstructive options for limb salvage around the periacetabular area include vascularized autografts; nonvascularized autografts; autoclaved, resected, or microwave-induced hyperthermia of bone from the same pelvis; massive pelvic allograft; endoprosthesis; resection arthrodesis; local improvised reconstruction with plates, pins and screws augmented with bone cement; and no reconstruction (internal hemipelvectomy) [2-16]. The average functional results following limb salvage in this particular area are fair and the complications for each reconstruction are not uncommon. We report the results of a patient with chondrosarcoma involving the whole pelvis and with contralateral involvement of the pubis, who was treated by an internal hemipelvectomy and by using polypropylene mesh graft for augmentation and reconstruction of muscles around the pelvis.

## Case presentation

A 45-year-old Thai male presented with a six-month history of right buttock and hip pain. The patient could not recall any injuries or incidents that may have caused the pain. He had no medical problems nor was he taking any medication. The MRI of the lumbosacral spine from the initial consultation at another hospital was unremarkable. He was treated unsuccessfully with a non-steroid anti-inflammatory drug and acetaminophen. He was referred to our institution for consultation. Physical examination revealed a healthy-appearing adult with an ill-defined 12 cm × 20 cm nontender firm mass at frank and groin area. There was no overlying skin change. Both hips had a full range of movement. The neurovascular function in the field of lower extremity was found to be intact, except the power of extensor hallucis longus on the right side was grade 4/5. He had no palpable lymphadenopathy. Laboratory studies were within normal limits.

**Figure 1:**
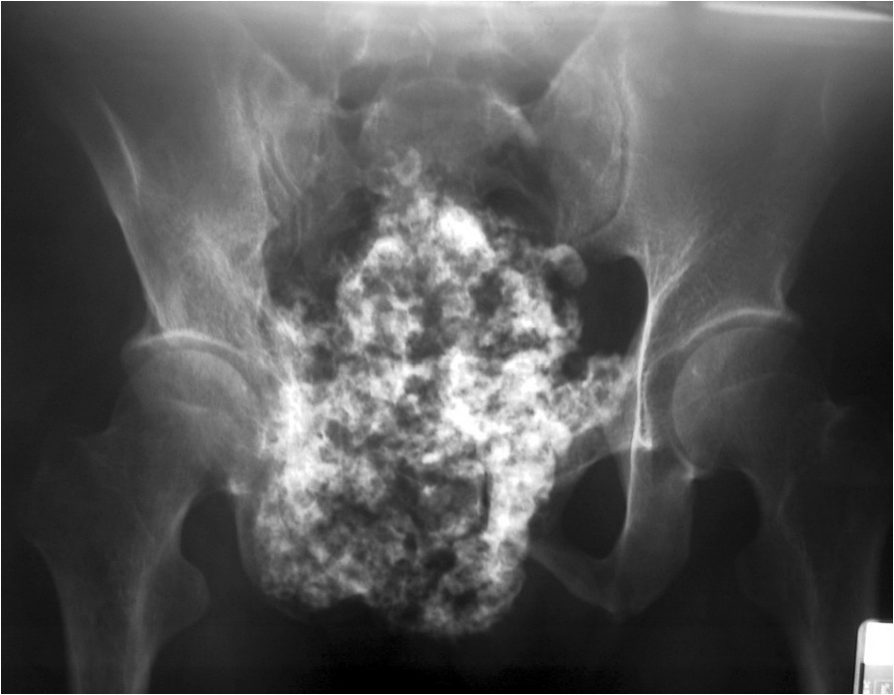
Anteroposterior radiograph of the pelvis showing chondrosarcoma is located at the right pubis and periacetabulum and extends to the left pubic area.

**Figure 2:**
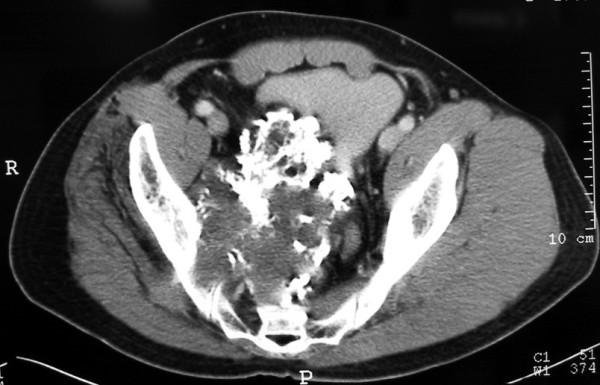
CT scan of the pelvis showing the tumor sized 12 cm × 10 cm originates from the right pelvis and invades the sacroiliac joint and pelvic cavity.

A plain radiograph of the pelvis revealed a well-defined mineralized mass extending from the right pubic and periacetabular area that also extended to the left pubic bone. The right obturator foramen was obliterated by the tumor mass. There was a popcorn-like chondroid matrix at the soft-tissue part of the tumor, which also extended into the pelvic cavity (Figure 1). Bone scintigraphy and computed tomography (CT) of the chest showed no distant metastasis except the abnormal uptake of the right pelvic and hip area. The CT scan of the pelvis showed a huge tumor mass in the pelvic cavity that involved the right sacroiliac joint and anterior border of the sacrum (Figure 2). The initial diagnosis was a chondrosarcoma involving both pubic areas, the right periacetabulum and ilium.

**Figure 3:**
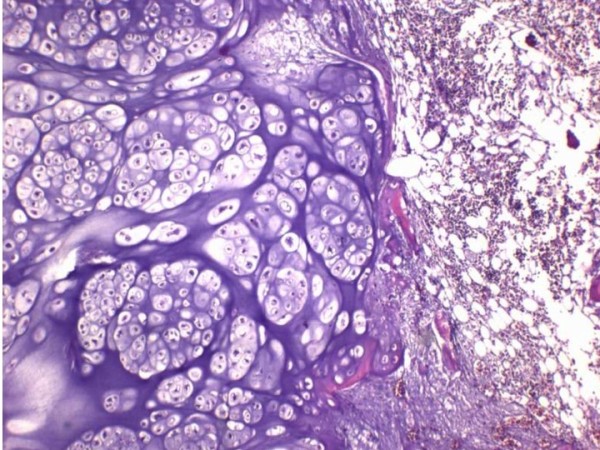
A photograph of the microscopic examination of the well-differentiated chondrosarcoma.

**Figure 4:**
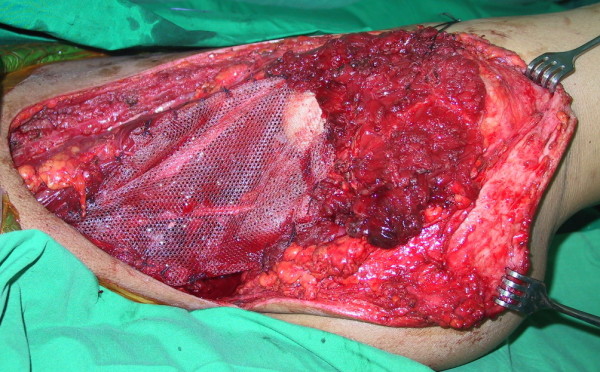
**An intraoperative photograph of the internal hemipelvectomy.** All dissected abdominal, back and gluteal muscles are sutured to the mesh.

**Figure 5:**
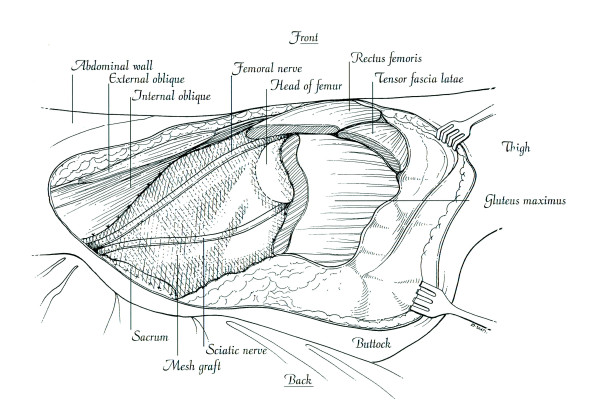
A drawing showing the intraoperative reconstruction of the patient.

**Figure 6:**
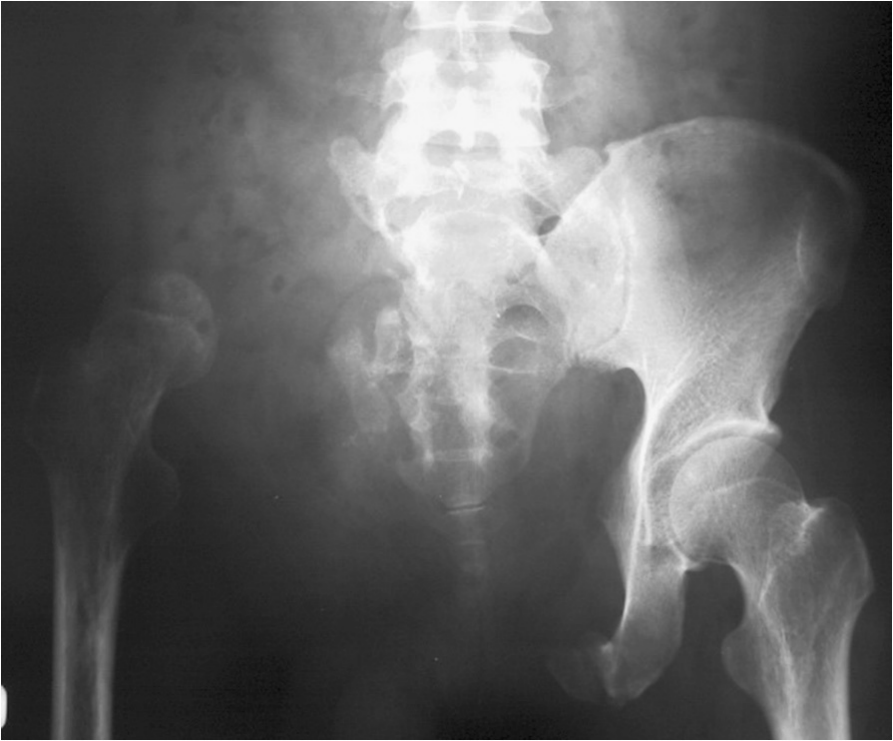
Anteroposterior radiograph of the pelvis postinternal hemipelvectomy showing an upward migration of the head of the right femur and tumor margin extending through the right sacral foramens.

Incisional biopsy was performed over the right pubic area. Microscopic examination revealed small chondrocytes with dark nuclei and scant cytoplasm. They were arranged in clones and binucleated cells were present. No mitotic figures were found. The diagnosis was consistent with chondrosarcoma. The tumor was classified as Grade I (well-differentiated) (Figure 3) according to the musculoskeletal tumor society staging system [17]. In this patient, the tumor resection was at PI-II-III, according to the Ennecking and Dunham classification [18] for the resection of primary tumor involving the pelvic bone. An internal hemipelvectomy with wide margin tumor removal was performed in this patient using the modified technique described by Eilber *et al*. [12]. The incision was made from left inguinal area to right pubis and anterior superior iliac spine, then curved superoposterior to iliac crest. The previous biopsy scar was ellipsed out from the incision. The external iliac vessels, femoral and sciatic nerves were preserved and mobilized from the tumor. The muscles attached to the pelvic bone were dissected out from the affected pelvis such as rectus abdominis, abdominal muscles, iliacus, gluteal muscles, rectus femoris, adductors and hamstrings. Osteotomies were made through the sacral ala just lateral to the neural foramina and contralateral pubic rami and ischium. A polypropylene mesh (Parietene, Sofradim, Trevoux, France) was used as an anchoring mesh to maintain all dissected muscle from the pelvis and soft-tissue reconstruction (Figure 4,5). All muscles were sutured along with mesh by prolene no. 0. They were kept attached with mesh at their optimal anatomical length to gain maximal muscle strength. Gluteal, lumbosacral and hamstrings were firstly sutured behind the mesh. Then, abdominal muscles, rectus abdominis, rectus femoris and adductor were sutured respectively by their anatomical layers. The final pathological report confirmed a well-differentiated chondrosarcoma with free margin. The radiograph of the pelvis following an internal hemipelvectomy showed upward migration of the head of the right femur (Figure 6).

**Figure 7:**
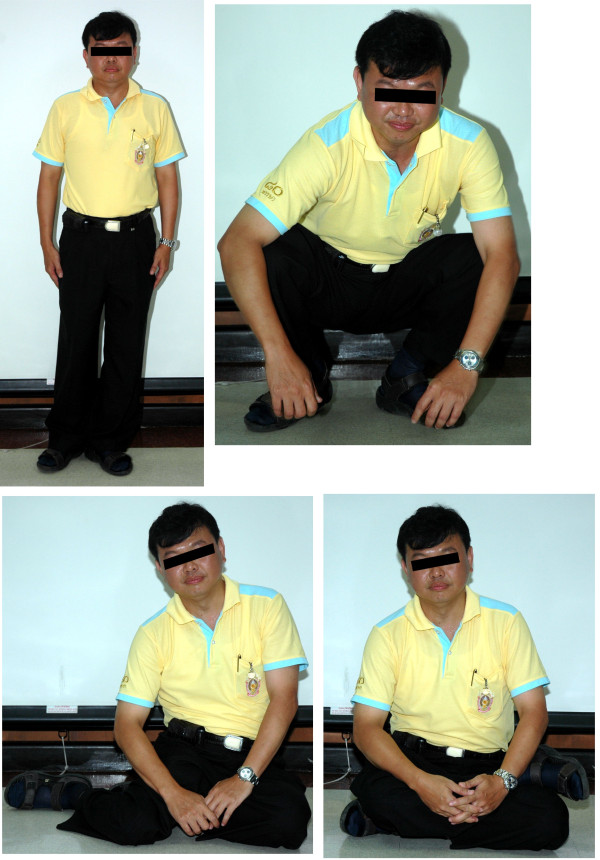
At postoperative 7.5 years, the patient is able to perform activities of daily living, including standing, walking, sitting and squatting.

Patient was placed in balanced skeletal traction at the tibial tuberosity with a weight of fifteen pounds for three weeks after surgery. Rehabilitation was uneventful. Progressive partial weight-bearing with axillary crutches was allowed after six to eight weeks. Regular strengthening exercises of the psoas, gluteus, quadriceps, and hamstring muscles were started and maintained to the highest level of tolerance during this period. At 7.5-year follow-up, the patient remained disease-free without local or distant relapse of the disease. No infection or wound complication occurred in this patient. Partial peroneal nerve palsy occurred, but was improved at the last visit. The patient had regained a range of motion of hip abduction of 15° and a range of hip flexion of 15°. The functional analysis at the final follow-up according to the Musculoskeletal Tumor Society system [19] was 66.7 %. He had a 3 cm leg-length discrepancy, which could be compensated by simple shoe lifts. He was able to perform most activities of daily living without any assistance. He could resume his employment as an electrical engineer by using one Canadian crutch during walking and could participate in sports exercise such as swimming or bicycling (Figure 7).

## Discussions

Primary malignant bone tumors involving the pelvis account for 15 % of all primary malignant bone tumors [1]. The goal of treating primary malignant bone tumors has expanded to both cure disease and provide a good quality of life. Tumors of the periacetabulum are amenable to curative intent but require wide or radical margins. Inadequate tumor surgical margin has a high risk of local recurrence and a poor prognosis for the patient. Classic hemipelvectomy is a common procedure for the removal of large tumors in the location that cannot be removed by limb salvage surgery with an adequate margin [4]. The rate of local recurrence was reported from 23 to 30 % [14,15,20,21]. This patient had an internal hemipelvectomy with a wide margin of tumor removal. Clear resection margin is the goal for chondrosarcoma, as there seems to be no convincing adjuvant treatment modality to prevent local recurrence in the event of tumor contamination. He remained disease-free without disease relapse at 7.5-year follow-up.

Several options for pelvic and periacetabular reconstruction after internal hemipelvectomy have been reported using vascularized autografts; nonvascularized autografts; autoclaved, resected, or microwave-induced hyperthermia of bone from the same pelvis; massive pelvic allograft; endoprosthesis; resection arthrodesis; local improvised reconstruction with plates, pins and screws augmented with bone cement; and no reconstruction (internal hemipelvectomy) [2-16]. Most reconstructive options for limb salvage in periacetabulum are associated with a high rate of complications and morbidity. The high complication rate is attributed to extensive bone loss and durability of the implant. The complications include deep infection, wound skin necrosis, and implant loosening. The rate of adjunctive surgery is high for the patients with such a complication [22,23]. Some patients even require implant or allograft removal that can result in a flail hip. Even a classic hemipelvectomy is determined in the final analysis for treating the complications. Several series demonstrated that internal hemipelvectomy without reconstruction in selective patients could provide fewer complications, acceptable function and cost-effectiveness of treatment [12,15,24].

For internal hemipelvectomy without reconstruction, surrounding muscles and soft tissue have been dissected from the resected pelvic bone tumor and need to be repaired and reconstructed to maintain the optimal function of the hip and leg for the patient. We used a Parietene polypropylene mesh (Parietene, Sofradim, Trevoux, France) as an anchoring material to re-approximate all the muscles that attach to the pelvis. All muscles including abdominal, gluteal, psoas, rectus femoris and hamstrings must be sewed back with a good anatomical length to gain optimal function [15]. With this technique and an optimal physical therapy program, the patient could gain acceptable function of the affected hip and leg and he could resume his previous profession. Asavamongkolkul *et al.* and Schwartz *et al.* demonstrated that patients with primary malignant bone tumor around the pelvic region had fair to good function with acceptable complication rates after tumor excision without reconstruction [15,16]. The patient in this report experienced a 3 cm leg-length discrepancy, he could compensate for by simple shoe lifting. He could resume his gainful employment and also enjoyed some sporting activities.

## Conclusions

Using polypropylene mesh graft for soft-tissue reconstruction in internal hemipelvectomy following malignant tumor removal is an alternative reconstruction in selected cases of pelvic malignant tumor. Patients undergoing this type of reconstruction, according to our prior experiences and from literature, can benefit from fewer complications with acceptable function.

## Consent

Written informed consent was obtained from the patient for publication of this case report and the accompanying images. Copies of the written consent form are available for review upon request.

## Competing interests

The authors declare that they have no competing interests.

## Authors’ contributions

AA composed the case report, prepared and edited this manuscript, contributed it conception, collected the data and conducted a literature search. SW participated in the data collection and gave final approval for this version of the manuscript. All authors read and approved the final manuscript.
